# From Monovalent EV-A71 Vaccines to Multivalent Vaccines Against Hand, Foot and Mouth Disease in China: Development Progress, Evidence Gaps, and Translational Challenges

**DOI:** 10.3390/vaccines14100842

**Published:** 2026-09-24

**Authors:** Yi Zhang, Yu Gu, Zhi Li, Bo Huang, Zhenzhuang Zou

**Affiliations:** 1 The First Clinical Institute, Xinpu Campus, Zunyi Medical University, Zunyi 563000, China; zy082059@zmu.edu.cn (Y.Z.); gy200236@zmu.edu.cn (Y.G.); lizhi879@zmu.edu.cn (Z.L.); 2 Department of Clinical Medicine, Zunyi Medical and Pharmaceutical College, Zunyi 563006, China

**Keywords:** hand, foot and mouth disease, enterovirus A71, multivalent vaccine, valency selection, translational evaluation

## Abstract

Hand, foot and mouth disease (HFMD) is caused by several enteroviruses, with enterovirus A71 (EV-A71) most strongly associated with severe disease and death. Licensed monovalent inactivated EV-A71 vaccines in China have a substantial evidence base for safety, immunogenicity, protective efficacy, and real-world effectiveness, together with relatively mature clinical evaluation and quality control systems. Their protection, however, is predominantly type-specific and does not extend reliably to major non-EV-A71 enteroviruses such as coxsackievirus A16 (CVA16), CVA6, or CVA10. As EV-A71-associated disease has declined, these viruses have become more prominent among residual cases and outbreaks, although shifts in etiological proportions should not be equated with vaccine-driven type replacement. This review synthesizes evidence on the developmental basis and limits of monovalent EV-A71 vaccination and evaluates multivalent candidates based on inactivated virus, virus-like particles (VLPs), chimeric antigens, and nucleic acid platforms. Most multivalent evidence remains preclinical, and major translational gaps include the absence of population-validated correlates of protection for non-EV-A71 types, uncertain antigenic coverage, component-specific quality control requirements, immune response imbalance, clinical case accrual, pediatric safety, coadministration, and affordability. We therefore propose a stage-gated framework that separates developmental maturity from public health priority and evaluates each additional antigen according to product objectives, sustained absolute disease burden, antigenic coverage, manufacturing readiness, immune response balance, clinical evaluability, and incremental public health value. Under this framework, higher valency is a conditional development choice rather than an objective in itself, and vaccine composition should be adapted to regional epidemiology and measurable added benefit over lower valency comparators.

## 1. Introduction

Hand, foot and mouth disease (HFMD) predominantly affects young children but can also occur in adults [1], and is caused mainly by viruses in the species *Enterovirus alphacoxsackie* [2], particularly enterovirus A71 (EV-A71), coxsackievirus A16 (CVA16), CVA6, and CVA10 [3,4,5]. Most cases are self-limiting, but EV-A71 can cause severe neurological and cardiopulmonary complications [3,4,6]. HFMD continues to impose a substantial disease burden in China, and the contributions of co-circulating enterovirus types vary markedly across regions and over time [7,8]. Historically, enterovirus types were defined by neutralization with type-specific antisera, whereas current typing relies mainly on VP1-based molecular methods [9]; however, serological characterization remains important for vaccine development because genetic relatedness does not necessarily predict antigenic equivalence or cross-neutralization. SCARB2 is a major receptor for EV-A71 and CVA16, whereas KREMEN1 functions as a receptor for CVA6 and CVA10; receptor usage informs viral entry and tropism but does not itself establish antigenic relatedness or vaccine cross-protection [10].

The poliovirus experience provides a useful precedent: monovalent oral poliovirus vaccines have been used for targeted control when a particular type predominates, whereas bivalent and trivalent formulations provide broader type coverage [11]. Because of its strong association with severe disease and death, EV-A71 was prioritized as the first vaccine target for HFMD [3,12]. Clinical trials established the safety, immunogenicity, and efficacy of inactivated EV-A71 vaccines under controlled conditions, whereas real-world studies demonstrated their effectiveness in routine use [13,14,15]. Their development also established precedents for type-specific clinical endpoints, neutralizing antibody assessment, reference standards, and potency testing [15,16,17,18,19]. However, protection remains predominantly type-specific, with no demonstrated prevention of HFMD caused by CVA16, CVA6, or CVA10 [20]. Following the introduction of EV-A71 vaccination in China, EV-A71-associated disease declined and non-EV-A71 enteroviruses accounted for a larger proportion of residual HFMD cases; this shift should not automatically be interpreted as vaccine-driven type replacement [14,21].

Previous reviews have summarized major challenges and platform progress in multivalent HFMD vaccine development [22,23], while a recent WHO/Medicines Patent Pool (MPP) technical meeting highlighted antigen selection, immune interference, reference materials, stability, and stepwise clinical evaluation [24]. However, these considerations have not been integrated into a type-specific decision framework, nor has it clearly distinguished developmental maturity from the public health rationale for each additional component [22,23,24]. This review focuses on the epidemiological context in China, while incorporating evidence from other settings when directly relevant. We assess the foundations and limitations of monovalent EV-A71 vaccines, update the development status of multivalent candidates, and propose a stage-gated framework for determining whether additional antigens should be incorporated. The framework links sustained absolute disease burden with antigenic coverage, manufacturing and quality-control readiness, component-specific immune-response balance, clinical evaluability, and incremental public-health value.

## 2. Literature Search and Evidence Selection

This article was conducted as a critical narrative review rather than a systematic review. PubMed was searched through 6 August 2026 for the literature on HFMD epidemiology, EV-A71 vaccine use, and multivalent HFMD vaccine development. Search terms included alternative virus name variants (“enterovirus A71,” “enterovirus 71,” “EV-A71,” “EV-71,” “EV71,” “coxsackievirus A16,” “CVA16,” “CV-A16,” “coxsackievirus A6,” “CVA6,” “CV-A6,” “coxsackievirus A10,” “CVA10,” and “CV-A10”) combined, as appropriate, with terms related to multivalent vaccines, vaccine platforms, neutralizing antibodies, correlates of protection, potency, and quality control.

ClinicalTrials.gov was searched for multivalent HFMD vaccine trials, and relevant WHO/Medicines Patent Pool technical materials and reference lists of relevant studies and reviews were also consulted. Retrieved records and reports were reviewed manually for relevance to the objectives of this review. Priority was given to evidence on the type-specific effectiveness and limitations of EV-A71 vaccines; the burden of non-EV-A71 enteroviruses; antigen and strain selection; multivalent vaccine development; immune interference; manufacturing and quality control; clinical endpoints and correlates of protection; and implementation and incremental public health value. Evidence from China was emphasized, with evidence from other settings included when directly relevant.

Candidate vaccine development status was assessed using publicly available information up to the final search date. Trial registration or a registry status of “completed” was not considered equivalent to published evidence of human immunogenicity or protective efficacy. Because this was a narrative review, evidence synthesis was qualitative; no formal risk-of-bias assessment or meta-analysis was undertaken.

## 3. Foundations of Monovalent EV-A71 Vaccine Development and Use

Among currently available HFMD vaccines, monovalent inactivated EV-A71 vaccines have the most mature technological pathway and the strongest clinical evidence base [12,13,15,25,26,27,28]. Their development therefore provides an important technical benchmark for the clinical evaluation, immunological assessment, and quality control of multivalent HFMD vaccines.

### 3.1. Target Selection and Basis for Clinical Evaluation

Although HFMD can be caused by multiple enteroviruses, EV-A71 is most strongly associated with severe disease and death and was therefore prioritized early in HFMD vaccine development [3,12,29]. This choice defined the principal public health objective of EV-A71 vaccination as the prevention of EV-A71-associated severe disease and death rather than all-cause HFMD [3,12,20,29]. A clearly defined target pathogen also simplified clinical evaluation: pivotal trials generally used laboratory-confirmed EV-A71-associated HFMD or other EV-A71-associated disease as primary endpoints, with EV-A71 neutralizing antibodies as the core immunological measure [15,16,25,26,27].

Multiple randomized, double-blind, placebo-controlled phase III trials have demonstrated the safety, immunogenicity, and type-specific protective efficacy of two-dose inactivated EV-A71 vaccines in young children [13,15,25,26,27]. A multicenter phase III trial published in 2026 enrolled 4011 children aged 2–71 months in Taiwan and Vietnam and further confirmed the clinical efficacy and safety of a bioreactor-produced inactivated EV-A71 vaccine, extending the evidence base beyond mainland China [30]. Importantly, this study evaluated a monovalent EV-A71 vaccine and therefore does not constitute human protection evidence for an EV-A71/CVA16 bivalent vaccine. Non-inferiority studies across EV-A71 products support the use of neutralizing antibodies for product comparison and immunobridging [28,31]; however, EV-A71 neutralizing antibody thresholds cannot be directly extrapolated as correlates of protection for vaccines targeting other enterovirus types [16,18,31,32].

### 3.2. Inactivated Vaccine Platform and Quality Control Experience

Major EV-A71 neutralizing epitopes depend largely on the three-dimensional conformation of the viral capsid proteins. Under appropriate inactivation and purification conditions, whole-virus inactivated vaccines can preserve viral particles and conformational antigens sufficiently to induce neutralizing antibody responses [12,33,34]. All EV-A71 vaccines licensed in China use a whole-virus inactivation platform, for which relatively mature manufacturing, quality control, and clinical evaluation systems have been established [12,17,18,19,28,31].

During EV-A71 vaccine development, methods for antigen content determination, neutralizing antibody testing, and reference standards were progressively established. The subsequent establishment of the WHO International Standard for human anti-EV-A71 serum further improved comparability across products and laboratories [17,18]. More recently, in vitro relative potency assays have shown good correlation with animal potency results, providing a potential route to reduce animal use and optimize lot release [19]. These experiences provide an important foundation for multivalent inactivated vaccines, although the ability to establish comparable component-specific evaluation systems for additional enterovirus types remains a key translational challenge.

### 3.3. Effectiveness and Boundaries of Protection

Following licensure, real-world studies supported the effectiveness of EV-A71 vaccines in reducing EV-A71-associated HFMD and severe disease [14,20,35,36]. In China, these vaccines remain outside the National Immunization Program and are distributed through the non-NIP market rather than being centrally funded [37].

Interpretation of real-world findings is nevertheless more complex than that of controlled clinical trials. HFMD epidemiology is shaped by multiple co-circulating enteroviruses, while vaccination coverage, changes in pathogen composition, epidemic cycles, and concurrent public health measures may all influence overall HFMD incidence [7,14,21,38]. Population-level evaluation of EV-A71 vaccination should therefore not rely solely on trends in clinically diagnosed HFMD, but should integrate virological surveillance and vaccination data to distinguish changes in EV-A71-associated disease from those caused by other enterovirus types.

The developmental pathway, evaluation framework, and limits of protection of monovalent EV-A71 vaccines are summarized in Figure 1.

## 4. Limitations of Monovalent EV-A71 Vaccines and the Rationale for Multivalent Vaccines

The rationale for multivalent HFMD vaccines depends on two interrelated questions: whether non-EV-A71 types cause sustained and measurable absolute disease burden, and whether the type-specific protection afforded by EV-A71 vaccination leaves a residual burden of public health importance [20,21,23,39]. Beyond epidemiology, multivalent vaccine development also requires consideration of antigenic coverage, manufacturing feasibility, immune response balance, and clinical evaluation [22,23,24,39]. In the framework proposed here, an additional enterovirus type should be incorporated only if it is expected to provide measurable added public health benefit over a lower-valency vaccine.

### 4.1. Persistent Circulation of Non-EV-A71 Enteroviruses in HFMD

HFMD outbreak surveillance in mainland China from 2011 to 2023 showed that, following the introduction of EV-A71 vaccination, the proportion of outbreaks attributed to EV-A71 declined from 40.43% to 3.07%, whereas the relative contributions of CVA16, CVA6, and other enteroviruses increased [21]. National surveillance from 2013 to 2019 and longitudinal studies in Guangdong and Shanghai likewise indicated that, as EV-A71-associated cases and severe cases declined, non-EV-A71 types did not decrease in parallel and continued to account for an important share of HFMD burden [21,35,40,41]. These findings indicate that EV-A71 vaccination effectively reduced disease caused by its target type without altering the fundamental multi-enterovirus ecology of HFMD.

CVA16 has long contributed to HFMD circulation and continues to spread across regions. Surveillance in Shenyang from 2013 to 2023 documented temporal shifts in dominant genetic lineages [42], while data from Hangzhou during 2021–2024 showed continued circulation of B1a and B1b lineages and an increase in the newly emerging B1c lineage in 2024 [43]. From a vaccine-development perspective, the relevance of CVA16 lies primarily in its persistent contribution to HFMD rather than the severity of any single outbreak. Although CVA16 is generally associated with milder disease, its sustained circulation supports its consideration as a candidate vaccine component. Ongoing lineage turnover also underscores the need to confirm that candidate strains provide adequate antigenic coverage.

Recent surveillance studies in China have identified a substantial contribution of CVA6 to typical and atypical HFMD and to clustered outbreaks, with detection also reported among severe cases [21,44,45,46]. National and local molecular surveillance further shows continued genetic evolution, recombination, and lineage replacement in CVA6 [44,46,47]. In Shandong Province in 2023, CVA6 accounted for 84.02% of laboratory-confirmed enterovirus-positive cases, illustrating its capacity to become locally predominant while continuing to evolve [46]. These observations support evaluation of CVA6 as an additional vaccine component in settings where a sustained and substantial absolute burden has been demonstrated, rather than its automatic inclusion in all trivalent formulations.

CVA10 is generally detected less frequently than CVA6 or CVA16, but it continues to be identified among patients with HFMD and herpangina, and a small number of severe cases have been reported [48,49,50,51]. Its inclusion in a tetravalent vaccine should therefore depend on sustained and measurable regional disease burden rather than sporadic detection. At present, evidence is insufficient to support routine inclusion of CVA10 across all regions of China.

Changes in etiological proportions require cautious interpretation. An increasing proportion of non-EV-A71 types does not, by itself, demonstrate vaccine-driven type replacement. Different enteroviruses have distinct epidemic cycles, and circulation is influenced by climate, contact patterns, population immunity, interactions among types, and viral evolution [3,7,52]. Moreover, even if the incidence of other types remains stable, a decline in the absolute number of EV-A71 cases will increase their proportional representation among residual cases [21,40]. Current evidence therefore indicates that non-EV-A71 types have become more prominent among residual HFMD cases, but does not establish stable or widespread vaccine-driven type replacement [14,21,40,52].

### 4.2. Type-Specific Protection Limits the Impact of Monovalent Vaccines on Overall HFMD Burden

Protection conferred by EV-A71 vaccines is largely restricted to EV-A71-associated disease. A 2024 real-world study found protection against EV-A71-associated HFMD but no detectable protection against cases associated with CVA16, CVA6, or CVA10 [20]. This limitation is consistent with antigenic differences in capsid structure and neutralizing epitopes among HFMD-associated enteroviruses [23,53,54]. Vaccine-induced antibodies can cross-neutralize different EV-A71 subgenotypes, but this activity does not reliably extend across distinct enterovirus types [20,23,55,56].

The effect of EV-A71 vaccination should therefore be distinguished at two levels: protection against EV-A71-associated HFMD and its effect on overall HFMD caused by all pathogens. The former is supported by substantial clinical trial and real-world evidence [13,14], whereas the latter also depends on the local pathogen distribution. In settings where EV-A71 accounts for a large share of disease, monovalent vaccination may substantially reduce overall burden; where non-EV-A71 types such as CVA6 or CVA16 predominate, its effect on total HFMD incidence is necessarily more limited [20,21,35,40,41,52].

EV-A71 vaccination was developed primarily to reduce severe disease and death, and its public health value for this objective is well established [12,13,14]. The broader burden of HFMD also includes mild disease, outpatient visits, family caregiving costs, and outbreaks in childcare settings [3,6,8,29,36,57,58,59]. Although substantial economic burden has been documented for HFMD in China, the available studies cited here did not estimate costs separately for EV-A71, CVA16, CVA6, and CVA10, limiting direct type-specific economic comparisons [57,58]. Accordingly, if the objective of vaccination is expanded to reduce all HFMD and clustered outbreaks rather than EV-A71-associated severe disease alone, non-EV-A71 types that cause sustained disease burden in the target setting must also be considered [20,21,23,39,53,59].

Figure 2 illustrates how type-specific protection against EV-A71 can reduce disease caused by the target type while leaving a variable residual burden attributable to other enteroviruses.

Taken together, the type-specific protection of monovalent EV-A71 vaccines and the residual burden caused by non-EV-A71 enteroviruses provide the principal rationale for multivalent HFMD vaccine development. However, an increase in the relative proportion of a type among residual cases does not mean that it should automatically be incorporated into a vaccine formulation. The translational value of any additional antigen should instead be judged according to sustained absolute disease burden, antigenic coverage, manufacturing and quality control feasibility, immune responses after combination, clinical evaluability, and incremental public health value.

## 5. Development Landscape of Multivalent HFMD Vaccines

Current multivalent HFMD vaccine candidates span whole-virus inactivation, VLPs, chimeric antigens, and nucleic acid platforms, with substantial differences in evidence maturity and translational barriers across approaches [23,56,60,61,62,63,64].

### 5.1. Valency Composition and Evidence Maturity of Candidate Vaccines

Current multivalent HFMD vaccine development focuses mainly on EV-A71, CVA16, CVA6, and CVA10 in bivalent, trivalent, and tetravalent combinations [23,60,61,62,63]. EV-A71/CVA16 bivalent vaccines have been studied most extensively; trivalent candidates commonly add CVA6, while tetravalent candidates further include CVA10 [60,61,62,65,66]. A CVA6/CVA10 combination without EV-A71 also provides evidence relevant to combined immunization against non-EV-A71 types and potential immune interference [67]. Overall, the published evidence for multivalent HFMD vaccines remains dominated by animal immunogenicity, neutralization, and challenge studies. A phase I/II trial of an inactivated EV-A71/CVA16 bivalent vaccine (NCT06063057) has been completed [68], and the candidate has progressed to phase III evaluation (NCT06734832) [69]. A phase I/II trial of an inactivated EV-A71/CVA16/CVA6/CVA10 tetravalent vaccine (NCT06754384) has also been completed [70], and a subsequent phase IIIa trial has been registered (NCT07611513) [71]. As of the final search date, no formally published human protective efficacy data were available for either multivalent inactivated candidate [68,69,70,71]. Current mRNA studies focus mainly on monovalent EV-A71 or CVA6 candidates, and no mature multivalent HFMD mRNA vaccine has yet been demonstrated; this platform is therefore not included in Table 1 [64,72]. Table 1 summarizes representative multivalent HFMD candidates by platform, key evidence, limitations, and development stage.

### 5.2. Multivalent Inactivated Vaccines

The whole-virus inactivation platform can build on the manufacturing and quality control experience established for EV-A71 vaccines and currently represents one of the more translationally mature approaches to multivalent HFMD vaccination. Nevertheless, a multivalent inactivated vaccine is not simply a mixture of monovalent antigens. Different enterovirus types may respond differently to inactivation and antigen preparation conditions, and antigen dose, adjuvant, and formulation must be optimized after combination [12,23,31,76,77,78].

Inactivated EV-A71/CVA16 bivalent vaccines have generated neutralizing antibody responses against both types and challenge protection against the corresponding virus in mouse and Mongolian gerbil models; based on animal neutralization and challenge results, several studies did not identify clear evidence of immune interference [60,66,73,74]. Repeat dose safety studies also suggest that a bivalent candidate is well tolerated in mice, although such findings cannot substitute for safety evaluation in the target pediatric population [79]. Trivalent inactivated vaccines have likewise generated preliminary evidence of immunogenicity against multiple enterovirus types and animal protection, but the number of studies and the extent of systematic comparison remain limited [61].

A major challenge for multivalent inactivated vaccines is that immune responses to individual antigen components may not be intrinsically balanced. In EV-A71/CVA16 bivalent formulations, the CVA16 component has shown a relatively weaker response after a single dose in some studies, with higher seroconversion after boosting, indicating that antigen-dose ratios, adjuvants, and immunization schedules can influence component-specific responses [78]. In addition, the effects of inactivation agents on the immunogenicity of CVA6, CVA10, and CVA16 are not uniform, and the inactivation process itself may alter capsid structure and surface properties [76,77]. Future development should therefore optimize inactivation conditions, antigen dose ratios, adjuvants, and immunization schedules for each component and establish component-specific potency and neutralizing antibody assays [23].

### 5.3. Virus-like Particle Vaccines

Virus-like particles are self-assembled viral structural proteins that can mimic the three-dimensional architecture of native capsids without containing a viral genome or having replicative or infectious capacity [80]. Compared with whole-virus inactivated vaccines, VLPs avoid large-scale propagation and handling of infectious virus and offer greater flexibility for antigen design, making them attractive for multitype combinations and epitope engineering [62,65,75]. Their immunogenicity, however, depends not only on antigen sequence but also on particle assembly and conformational stability [80,81]. Structural studies indicate that recombinant CVA16 VLPs can retain a structure and antigenicity resembling native empty particles, while particle expansion or other conformational changes may still alter the presentation of neutralizing epitopes [81]. This dependence on structural integrity represents an important translational distinction between VLP and conventional inactivated platforms.

Current studies provide preliminary support for the preclinical feasibility of multivalent HFMD VLP vaccines. An EV-A71/CVA16 bivalent VLP vaccine induced neutralizing antibodies against both types, and passive transfer of immune serum protected against challenge with either virus [65]. A tetravalent VLP vaccine covering EV-A71, CVA16, CVA6, and CVA10 likewise elicited neutralizing antibodies and animal protection against all four types, suggesting that broad type-specific responses can be maintained after combining multiple VLP antigens [62]. Nevertheless, these findings are derived entirely from animal models and do not establish immune response balance or clinical protection in children.

Monovalent VLP studies targeting non-EV-A71 types also provide evidence relevant to antigen selection for multivalent vaccines. A CVA6 VLP based on the predominant D3 genotype circulating in China elicited neutralizing antibodies against D3a and D3b lineages and conferred protection in active and passive immunization models, suggesting potential within-type coverage across genetic lineages [82]. Such studies can inform whether a candidate antigen covers major circulating lineages, but they do not replace evaluation of component-specific immune balance and formulation stability in a multivalent product. Chimeric VLPs and epitope engineering strategies may also broaden antigenic coverage, although their evidence base and limitations are discussed separately below.

Further translation of VLP vaccines depends on the maturity of manufacturing and quality-evaluation systems. Different enterovirus VLPs require distinct ratios of structural proteins to viral proteases, culture conditions, and purification processes. Recent work showed substantial differences in optimal production conditions and purification recovery between EV-A71 and CVA6 VLPs, indicating that each component may require separate optimization [80]. For multivalent formulations, particle integrity, antigen ratios, formulation stability, and lot-to-lot consistency must also be controlled, alongside assays capable of simultaneously measuring type-specific neutralizing antibodies [83].

### 5.4. Chimeric Antigens and Emerging Vaccine Platforms

Rather than adding multiple complete antigens to a single formulation, chimeric antigen approaches seek to display neutralizing epitopes from different enterovirus types within a common particle or expression scaffold. Early studies displaying a CVA16 epitope on an EV-A71 VLP generated antibodies against both types and provided animal protection [75]. Subsequently, CVA16 and CVA10 epitopes were introduced by replacing surface loops on an EV-A71 VLP. Although the resulting chimeric VLP induced antibodies against all three viruses, protection rates after challenge were only 20% against CVA16 and 40% against CVA10 [56]. These findings illustrate that immunological recognition of an epitope does not guarantee that it retains an effective neutralizing conformation on a heterologous scaffold. Epitope selection, insertion site, and the structural consequences for the VLP are therefore central determinants of whether chimeric vaccines can broaden protection.

DNA vaccines translate chimeric antigen and epitope combination concepts into a nucleic acid expression platform. A tetravalent DNA candidate based on EV-A71 VP1 and incorporating multiple neutralizing epitopes from EV-A71, CVA16, CVA6, and CVA10 induced antibody and CD8^+^ T-cell responses in mice [63]. However, the study focused primarily on immunogenicity and linear epitope responses, and did not establish that the induced antibodies could independently neutralize all four viruses or provide multitype challenge protection. This candidate therefore represents an early validation of tetravalent antigen design and immunogenicity rather than complete evidence of multitype protection.

mRNA-based HFMD vaccine research is at an earlier stage. A nucleoside-modified EV-A71 mRNA vaccine induced humoral and cellular immunity in mice and conferred protection after both active immunization and immune serum transfer [64]. An mRNA vaccine based on the CVA6 D3a subgenotype also elicited strong T-cell responses and showed cross-protection against CVA10 infection in mice [72]. However, these studies used EV-A71 or CVA6 as single antigen sources and therefore demonstrate the feasibility of the mRNA platform for monovalent HFMD-related antigens rather than validation of a multivalent HFMD mRNA vaccine.

Chimeric antigen and nucleic acid platforms should therefore currently be regarded as exploratory approaches. Their potential advantage lies in the ability to broaden coverage using fewer antigen modules and to provide technical flexibility for future adjustment of vaccine composition as pathogen patterns evolve. At present, however, their translation is constrained by uncertainty regarding stable presentation of conformational epitopes, balanced neutralizing responses across types, and reproducible multitype challenge protection.

## 6. Discussion: A Proposed Stage-Gated Framework and Key Evidence Gaps for Multivalent HFMD Vaccines

Multivalent HFMD candidates have generated evidence of immunogenicity against multiple enterovirus types, neutralizing antibodies, and protection in animal challenge models [60,61,62,63,66,67,73,74]. These findings primarily show that multiple antigens can be combined and can induce immune responses; they do not demonstrate that a higher valency vaccine will necessarily provide greater clinical or public health value. Translational evaluation must also establish whether an additional type causes a sustained and preventable disease burden, whether the candidate antigen covers major circulating lineages, whether each component remains functional after combination, and whether broader antigenic coverage translates into additional reductions in disease burden under real-world vaccination conditions.

Accordingly, the commonly described progression from bivalent to trivalent and then tetravalent formulations should be viewed as one possible development pathway rather than a universal rule. We therefore propose a conceptual stage-gated framework (Table 2) integrating product objectives and target populations, epidemiological need, antigenic coverage, manufacturing and quality control, immune response balance, clinical evaluability, and incremental public health value. The framework is intended to support comparative assessment of candidate antigens and development decisions; it has not undergone formal expert consensus, regulatory evaluation, or external validation.

### 6.1. Product Objectives and Target Populations

The antigen composition of a multivalent HFMD vaccine should first be determined by its predefined product objectives. If the primary objective is to reduce neurological complications, severe disease, and death, EV-A71 should generally remain a core component because of its strong association with severe clinical outcomes and the relatively mature clinical evaluation and quality control systems established for EV-A71 vaccines [3,12,13,15,17,18,19]. If the objective is expanded to reduce all laboratory-confirmed HFMD or outbreaks in childcare settings, non-EV-A71 types that cause sustained disease burden in the target population must also be evaluated [12,15,59]. The same antigen may therefore have a different priority depending on the intended product profile.

Early development should also define whether a multivalent vaccine is intended to replace existing monovalent EV-A71 vaccines, to immunize vaccine naive children, or to serve as a booster in previously vaccinated children. Target age, vaccination schedule, comparator formulation, and primary outcomes should be prespecified so that the clinical and public health value of an additional component can be interpreted appropriately.

### 6.2. Epidemiological Need and Valency Prioritization

The epidemiological priority for adding an enterovirus type should be based primarily on sustained disease frequency and severity rather than economic considerations at this stage. No validated numerical thresholds currently exist for prioritizing additional HFMD vaccine antigens. National surveillance data indicate that vaccine-relevant enterovirus types can contribute on the order of 10% or more of type-identified HFMD cases and 5% or more of type-identified severe cases during epidemic periods [94,95]. On this basis, and for research prioritization only, we regard approximately 10% of type-identified HFMD cases and approximately 5% of type-identified severe cases as useful working benchmarks for frequent circulation and clinically relevant severe disease burden, respectively. These values should be regarded as approximate benchmarks rather than fixed inclusion criteria and should be interpreted together with absolute incidence, persistence across epidemic seasons, regional variation, and surveillance quality. A transient increase in proportional detection or a single localized outbreak alone would not justify long-term inclusion in a vaccine formulation.

EV-A71 should remain a foundational component of formulations whose primary objective is to prevent severe disease and death. CVA16 has shown a sustained contribution to HFMD burden and has already been combined with EV-A71 in both inactivated and VLP candidates, with a substantial preclinical evidence base and clinical development of a bivalent inactivated vaccine [60,65,68,69,94]. This gives CVA16 a relatively advanced translational position, although developmental maturity alone should not determine public health priority. Where surveillance across multiple epidemic seasons demonstrates a sustained burden of typical or atypical HFMD, outbreaks, or severe disease attributable to CVA6, this type may warrant higher regional priority for evaluation [21,40,46,47,95]. By contrast, the burden attributable to CVA10 has generally been smaller and more variable [23,48,50]. Its inclusion should therefore depend on longitudinal evidence of sustained and clinically meaningful disease burden rather than on an objective of increasing vaccine valency alone.

Regional prioritization should not rely on local epidemiology in isolation, because population mobility can facilitate interregional spread; surveillance and vaccine composition decisions should therefore also consider epidemiologically connected regions and the potential for pathogen importation [96].

### 6.3. Antigenic Coverage and Candidate Strain Suitability

Once the epidemiological need for a type has been established, candidate antigens should be evaluated for their ability to cover contemporary circulating viruses. Candidate strains should be tested against representative isolates and major circulating lineages, with attention to within-type cross-neutralization, assay virus selection, and the effects of viral particle conformation [55,81,82,87,88]. Type designation alone is not a substitute for evidence of antigenic coverage.

Different EV-A71 subgenotypes show broad cross-antigenicity, although neutralizing antibody titers can still vary [55]. A CVA6 VLP based on the predominant D3 genotype in China induced neutralizing antibodies against D3a and D3b lineages and provided animal protection, offering preliminary evidence of within-type coverage [82]. Neutralization results for both CVA6 and CVA10, however, can depend on the assay strain selected [87,88]. A high neutralizing titer against a single laboratory strain therefore does not establish adequate coverage of circulating viruses. Beyond cross-neutralization breadth, in vitro escape-selection studies may provide complementary information on antigenic robustness by identifying mutations that reduce antibody neutralization [33,97]. Future studies should therefore combine prespecified panels of representative isolates with, where appropriate, escape-selection analyses and should reassess candidate strain suitability as dominant lineages change. However, no standardized escape-frequency threshold has been established for HFMD vaccine strain selection, and such testing should complement rather than replace cross-neutralization against representative circulating isolates.

### 6.4. Manufacturing and Component-Specific Quality Control

Even a type with high epidemiological priority may need to be deferred if manufacturing or quality control readiness is inadequate. For inactivated EV-A71 vaccines, antigen and neutralizing antibody reference standards and in vitro relative potency methods have already been established [17,18,19], but these systems cannot simply be transferred to other enterovirus types.

Different enteroviruses respond differently to culture, purification, and inactivation conditions. For CVA6, formaldehyde and beta-propiolactone inactivation have produced similar immunogenicity, whereas beta-propiolactone has generated stronger immune responses than formaldehyde for CVA10 and CVA16 [76]. Beta-propiolactone may also alter the capsid conformation and surface properties of CVA16 [77]. These findings demonstrate that meeting a total antigen content specification does not guarantee preservation of critical neutralizing epitopes and that a single inactivation condition or total antigen metric cannot be assumed to be appropriate for every component.

Multivalent formulations must also demonstrate that each component remains functionally intact after mixing, adjuvant adsorption, and storage. Antigen binding to aluminum adjuvants and antigen stability depend on both antigen properties and formulation conditions [98,99], while the distribution of multiple antigens on adjuvant particles may depend on the mixing process [100]. Component-specific identity, content, conformational integrity, potency, stability, and lot-to-lot consistency should therefore be evaluated [83,89,98]. Platform-specific attributes must also be considered: VLP vaccines require assessment of particle assembly, chimeric antigens require confirmation of epitope exposure, and nucleic acid vaccines require consistent antigen expression [63,64,72,80].

### 6.5. Immune Response Balance and Dose Optimization

The ability of a multivalent formulation to induce several neutralizing antibody responses does not ensure that every component retains adequate immunogenicity after combination. Studies of EV-A71/CVA16 bivalent vaccines suggest that the CVA16 component may produce a relatively weaker neutralizing response after a single dose, with a clearer increase after boosting [66,73,78]. Multivalent formulations should therefore be compared directly with the corresponding monovalent or lower valency vaccines for component-specific antibody titers, seroconversion, dose–response relationships, and animal protection; in human studies, component-specific non-inferiority criteria should be prespecified [28,31].

Several trivalent and tetravalent preclinical studies have not identified obvious immune interference, but antigen doses, adjuvants, animal models, and assay methods differ across studies [23,39,61,62]. These findings do not establish that adding CVA6 or CVA10 will leave EV-A71 or CVA16 responses unaffected, nor can they be directly extrapolated to young children. Antigen dose ratios in multivalent formulations should not be assigned as equal quantities solely on the basis of component count; instead, they should be optimized according to component-specific immunogenicity, stability, and antibody persistence. If an additional component requires impractically high antigen doses or a substantially more complex schedule to achieve an adequate response, formulation optimization should precede progression to a higher valency.

### 6.6. Correlates of Protection and Clinical Evaluation Strategy

For EV-A71 vaccines, population-level evidence links neutralizing antibody responses with clinical protection. Previous studies have associated a neutralizing antibody titer of 1:16 with protection against EV-A71-associated disease [26]; other analyses have suggested protective levels around 1:16–1:32, or a day-56 concentration of 14.7 U/mL corresponding to approximately 50% protection over the subsequent year [16,32]. These estimates depend on assay systems, follow-up duration, and study populations and should not be extrapolated without regard to their methodological context.

Comparable population-level protection data are not yet available for CVA16, CVA6, or CVA10. Current evaluation relies mainly on neutralization assays, neonatal mouse challenge models, and passive transfer of immune serum [60,61,62,66,73,74,76]. These studies support antibody-mediated protection in animal models but do not define the antibody level required to reduce disease risk in vaccinated children. Neutralizing antibodies against non-EV-A71 types should therefore currently be regarded primarily as immunogenicity measures rather than direct substitutes for clinical protection endpoints. From a regulatory perspective, the absence of validated correlates of protection for CVA16, CVA6, and CVA10 limits the evidentiary basis for using neutralizing antibody responses as stand-alone surrogate endpoints. WHO guidance for EV-A71 indicates that the absence of an established correlate does not necessarily preclude immunobridging, but such an approach depends on comparison with an efficacious licensed vaccine containing the same antigen(s) and on directly comparable immune responses [101]. A four-color pseudovirus neutralization assay can simultaneously evaluate antibodies to the four major types [83], but further validation with clinical samples and interlaboratory studies is required.

Clinical evaluation should use pathogen-confirmed, type-specific endpoints. A pooled endpoint comprising laboratory-confirmed HFMD caused by all vaccine-covered types may improve case accrual, but type-specific analyses should be retained so that a more common type does not mask inadequate protection from an added component [15,20,26,27]. Because the incidence of individual enterovirus types varies substantially across regions and years [21,29,40], multivalent vaccine trials should therefore use multicenter designs spanning multiple epidemic seasons, with a prespecified hierarchy among overall efficacy, pooled efficacy against covered types, and efficacy for each component. For CVA16, CVA6, and CVA10, no analogous efficacy-established licensed comparator is currently available; therefore, immunobridging for these components remains less straightforward, and pathogen-confirmed, type-specific efficacy data may remain necessary during pivotal development. Prospective serum sampling linked to virologically confirmed outcomes could simultaneously help establish type-specific protection relationships and strengthen the basis for future immunobridging [16,31,102].

### 6.7. Pediatric Safety, Coadministration, and Implementation

Adding antigen components may alter the safety and reactogenicity profile of a vaccine. Multivalent formulations may involve changes in total antigen content, adjuvant dose, formulation interactions, or immunization schedule; local reactions, systemic reactions such as fever, and serious adverse events should therefore be systematically evaluated in the target pediatric age group [31]. Repeat dose safety evidence for current EV-A71/CVA16 bivalent candidates is derived mainly from animal studies [79] and cannot substitute for pediatric clinical evaluation.

Maternally derived antibodies, prior natural infection, and previous EV-A71 vaccination can produce different baseline antibody profiles [84,85,86]. Clinical studies should therefore prespecify analyses of vaccine naive children, children previously vaccinated against EV-A71, and participants with different baseline antibody levels to avoid misinterpreting pre-existing immunity as evidence of relative strength or weakness of a vaccine component.

A randomized controlled study has evaluated the immunogenicity and safety of coadministering a monovalent EV-A71 vaccine with hepatitis B vaccine, group A meningococcal polysaccharide vaccine, measles-rubella vaccine, or Japanese encephalitis vaccine [93]. These findings cannot be directly extrapolated to antigenically more complex multivalent HFMD vaccines. Future studies should assess both safety and immune responses to each HFMD component and to routine childhood vaccines under coadministration schedules, as well as compatibility with existing immunization programs. At present, EV-A71 vaccination in China is not included in the National Immunization Program and is administered on a voluntary, self-paid basis [37]. Therefore, no routine national schedule yet exists for a future multivalent HFMD vaccine; if such a vaccine is introduced into routine childhood immunization, its age window and coadministration strategy should be evaluated specifically to minimize additional visits while preserving immune responses to both HFMD components and scheduled childhood vaccines [37,93].

### 6.8. Incremental Public Health Value

The value of a multivalent vaccine should be judged relative to monovalent EV-A71 vaccination or lower-valency alternatives rather than by the number of enterovirus types included. Multicity surveillance in China indicates that target-type disease declined after EV-A71 vaccine introduction, but the magnitude of real-world impact varied with regional pathogen burden and achieved vaccination coverage [14,21,40]. The benefit of adding a component therefore depends not only on its type-specific vaccine effect but also on the absolute burden caused by that type in the target population.

Economic considerations are most appropriately applied at the implementation stage rather than as primary criteria for initial antigen selection. Price and willingness to vaccinate may further modify real-world impact. A parental survey found that vaccine price acceptability and recommendations from healthcare professionals were associated with willingness to vaccinate [92]. Economic models likewise indicate that the advantage of an EV-A71/CVA16 bivalent vaccine over a monovalent vaccine varies with vaccine price, CVA16 disease burden, duration of protection, and vaccination coverage [90,91]. However, available economic burden studies in China have generally reported costs by disease severity, healthcare use, or region rather than by enterovirus type, so direct economic comparisons among EV-A71, CVA16, CVA6, and CVA10 remain limited [57,58]. If higher manufacturing and component-specific testing costs substantially increase the price of a multivalent product, gains from broader theoretical coverage may be partly offset by lower uptake.

Post-licensure evaluation should link vaccination records with case surveillance and virological typing. Overall HFMD incidence is sensitive to epidemic cycles, population mobility, patterns of contact among children, and surveillance intensity [7,14,38,40,96], making total case trends difficult to attribute to vaccination alone. Absolute incidence of types covered and not covered by the vaccine should be monitored separately, together with outcomes such as outpatient visits, hospitalization, severe disease, and clustered outbreaks.

Valency selection should therefore be treated as an iterative, evidence-responsive process rather than a fixed one-way progression from monovalent to higher-valency formulations. An additional component should progress to later development or broader use only when it satisfies epidemiological, antigenic, manufacturing, immunological, clinical, and implementation criteria and provides measurable additional health benefit over a lower-valency comparator.

## 7. Conclusions

Monovalent inactivated EV-A71 vaccines demonstrate that type-specific immunization against a major cause of severe HFMD can deliver clear public-health benefit, but they also illustrate the limitations of a single-antigen strategy for a disease caused by multiple co-circulating enteroviruses. Multivalent vaccines are therefore a rational next step, yet broader antigenic coverage alone does not guarantee greater health benefit. Their value depends on whether an added type causes a sustained and preventable disease burden and whether the corresponding antigen remains stable, immunologically balanced, and clinically evaluable within a combination formulation. At present, EV-A71/CVA16 bivalent vaccines are closest to clinical translation; CVA6 may provide additional value in selected high-burden settings, whereas evidence remains insufficient to support routine inclusion of CVA10. Future multivalent HFMD vaccine composition should be adapted dynamically to epidemiological change and translational evidence rather than driven by an objective of maximizing valency. Given the narrative design and the absence of a formal risk-of-bias assessment, these conclusions should be interpreted with appropriate caution. A multivalent strategy should be considered superior to an existing monovalent or lower-valency vaccine only when its additional components produce measurable reductions in disease burden under real-world vaccination conditions.

## Figures and Tables

**Figure 1:**
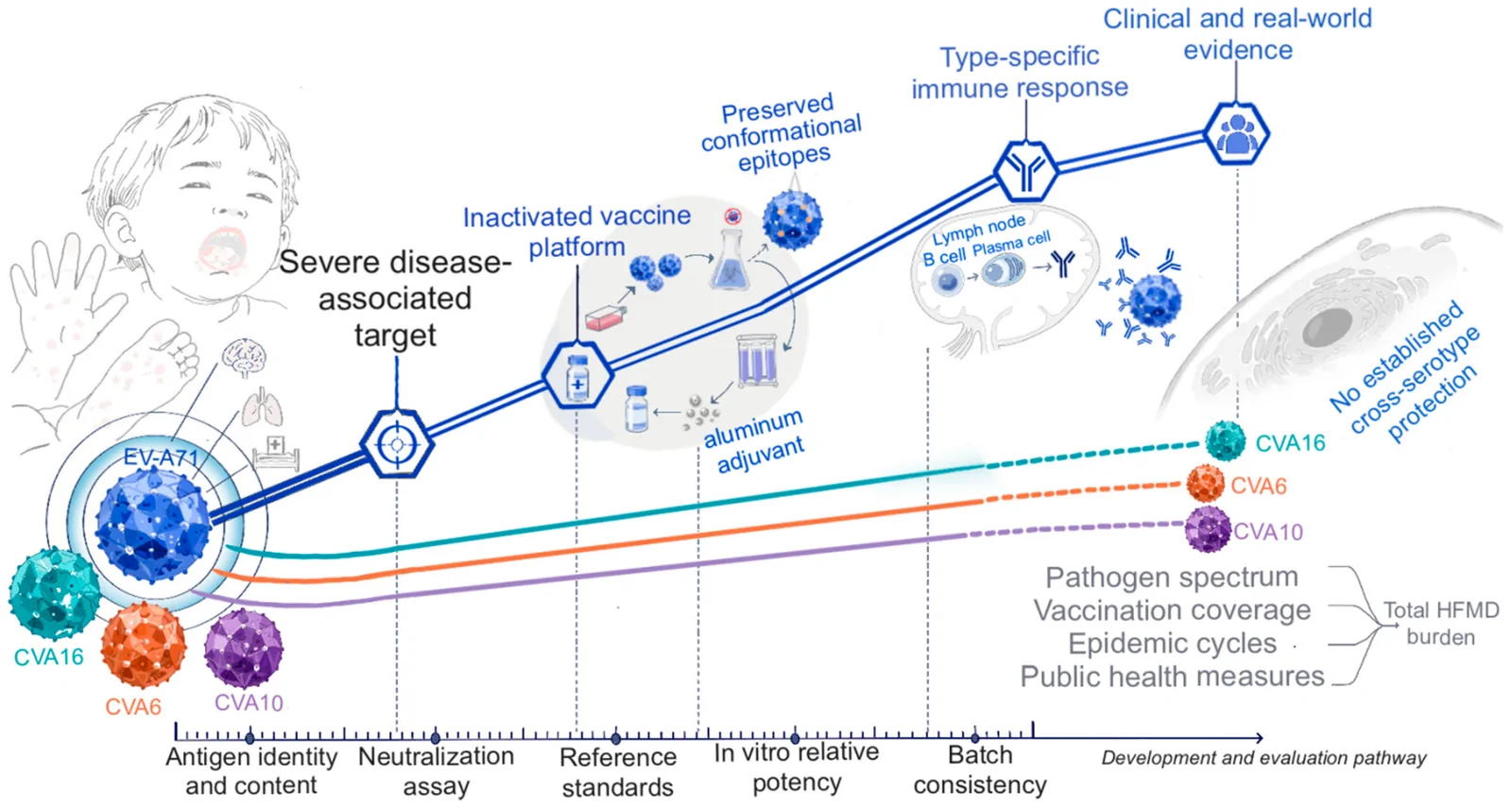
Developmental pathway and limits of protection of monovalent inactivated EV-A71 vaccines. The blue pathway summarizes progression from selection of EV-A71 as a severe-disease target through vaccine production, preservation of conformational epitopes, type-specific immune responses, and clinical and real-world validation. The lower sequence shows major evaluation steps, including antigen identity and content, neutralization assays, reference standards, in vitro relative potency, and batch consistency. Cyan, orange, and purple lines represent CVA16, CVA6, and CVA10, respectively, for which cross-type protection by monovalent EV-A71 vaccination has not been established. Horizontal position indicates developmental sequence rather than a quantitative axis or timescale.

**Figure 2:**
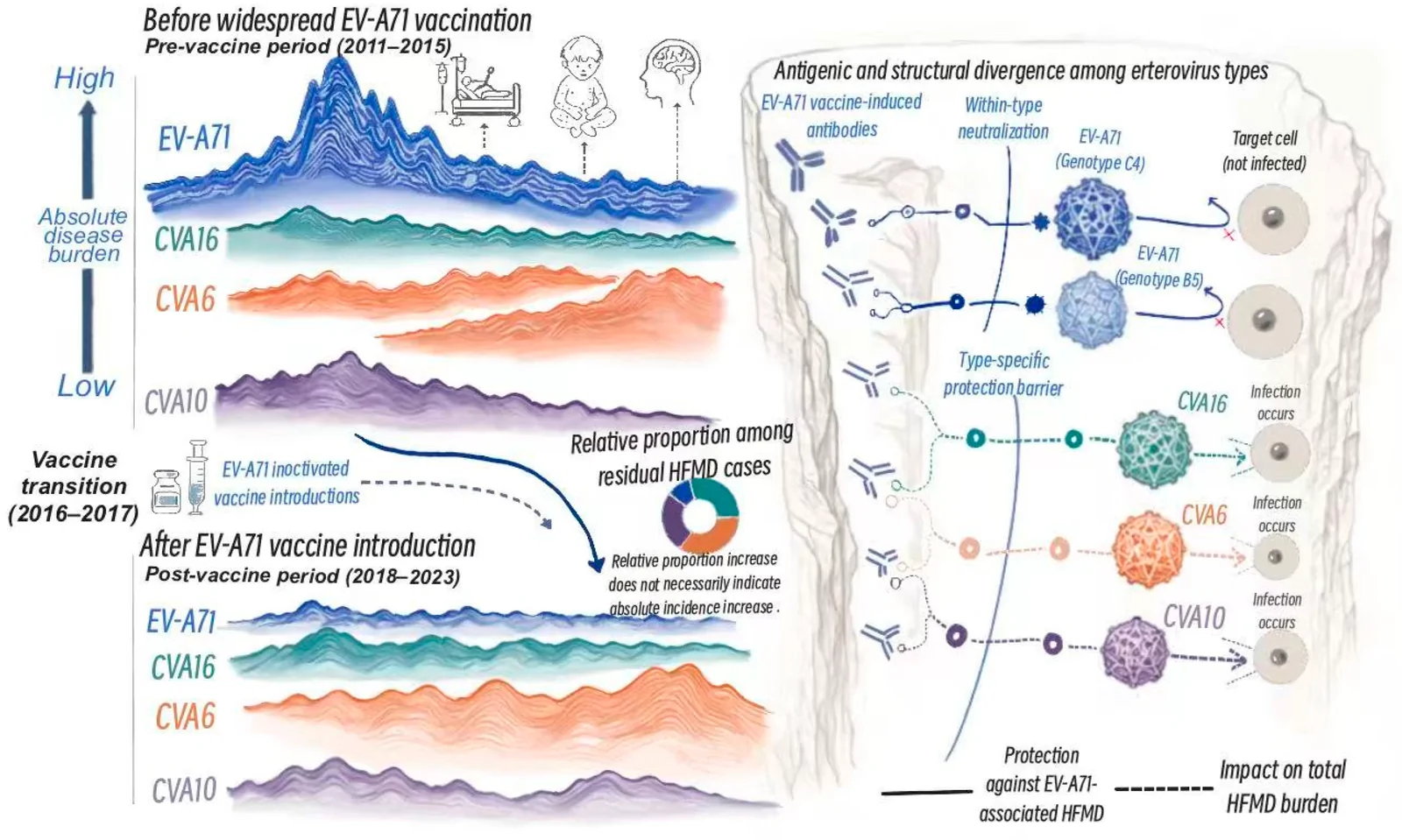
Type-specific EV-A71 protection and residual HFMD burden in China. The left panel schematically compares the pre-vaccine (2011–2015), vaccine-transition (2016–2017), and post-vaccine (2018–2023) periods defined in national outbreak surveillance [21]. The wave-like profiles are conceptual and illustrate changes in the relative contribution of EV-A71, CVA16, CVA6, and CVA10 rather than measured annual incidence. The gray dashed arrow denotes the transition following EV-A71 vaccine introduction to the post-vaccine period, whereas the solid blue arrow indicates the shift in the relative composition of residual HFMD cases. The inset emphasizes that an increased relative proportion of a non-EV-A71 type does not necessarily indicate increased absolute incidence. The right panel illustrates within-type neutralization among EV-A71 genotypes and the limited cross-type protection against CVA16, CVA6, and CVA10. The two panels jointly illustrate why EV-A71 vaccination can reduce EV-A71-associated HFMD while leaving residual disease caused by other enterovirus types. EV-A71, enterovirus A71; HFMD, hand, foot and mouth disease.

**Table 1:** Representative multivalent HFMD vaccine candidates: technology platforms, key evidence, limitations, and development stage.

Candidate/Platform	Key Evidence and Development Status	Key Limitations	Evidence Maturity/ Supporting Studies
**Bivalent EV-A71/CVA16 inactivated vaccine** *Whole-virus inactivated vaccine*	Induces type-specific neutralizing antibodies against EV-A71 and CVA16. Active immunization or passive transfer of immune serum provides type-matched challenge protection in mouse or Mongolian gerbil models [60,66,73,74].	Antigen doses, adjuvants, and animal models vary across studies. Some studies report weaker CVA16 responses after a single dose. Human protective-efficacy data are not yet published.	4 published preclinical studies; phase I/II completed; phase III ongoing
**Bivalent CVA6/CVA10 vaccine** *Whole-virus inactivated vaccine*	Induces type-specific neutralizing antibodies against CVA6 and CVA10. Protection was demonstrated in actively immunized mice and in antibody-transfer and maternal-antibody models [67].	Evidence is dominated by a single mouse study. Because EV-A71 is absent, this formulation mainly informs the feasibility of combining non-EV-A71 antigens.	1 published preclinical study; proof of concept
**Bivalent EV-A71/CVA16 VLP vaccine** *VLP vaccine*	Induces relatively balanced neutralizing antibody responses to both serotypes. Passive transfer of immune serum protects mice against lethal challenge with EV-A71 or CVA16. The formulation was also compared with the corresponding monovalent VLPs [65].	Evidence is limited to mouse models. Human immunogenicity, durability, scalable manufacturing, and lot-to-lot consistency remain uncharacterized.	1 published mouse study; proof of concept
**Bivalent EV-A71/CVA16 chimeric VLP vaccine** *Chimeric VLP*	Presents a CVA16 neutralizing epitope on an EV-A71 VLP scaffold and induces antibodies specific to both serotypes. Challenge studies in mice also provided evidence of protection against both serotypes [75].	Insertion site, heterologous-epitope conformation, strain coverage, and scalable manufacturing require further validation.	1 published mouse study; early proof of concept
**Trivalent EV-A71/CVA16/CVA6 vaccine** *Whole-virus inactivated vaccine*	Induces neutralizing antibodies against all three serotypes. Active immunization protects against EV-A71 and CVA16 challenge, while passive transfer of trivalent immune serum protects against CVA6 challenge [61].	Few studies are available, and protection across the three types has not been assessed with fully harmonized methods. Dose ratios, durability, and comparisons with lower-valency formulations remain insufficient.	1 published mouse study; proof of concept
**Tetravalent EV-A71/CVA16/CVA6/CVA10 inactivated vaccine** *Whole-virus inactivated vaccine*	The phase I/II trial has been completed (NCT06754384), and a subsequent phase IIIa trial has been registered (NCT07611513) [70,71].	No human immunogenicity or protective-efficacy results have been formally published. Component-specific balance and durability remain to be reported.	2 registered clinical trials; no published human results; phase I/II completed; phase IIIa registered
**Tetravalent EV-A71/CVA16/CVA6/CVA10 VLP vaccine** *VLP vaccine*	Induces broad and relatively balanced neutralizing antibody responses to all four viruses. Animal studies also provided evidence of challenge protection against all four serotypes [62].	Evidence is dominated by a single mouse study. Human data, durability, dose optimization, scalable manufacturing, and lot-to-lot consistency are lacking.	1 published mouse study; proof of concept
**Tetravalent EV-A71/CVA16/CVA6/CVA10 DNA vaccine** *Multi-epitope DNA vaccine*	Induces antibody and CD8^+^ T-cell responses in mice; immunodominant linear B-cell epitopes were also identified [63].	Neutralizing-antibody responses against all four viruses and multitype challenge protection have not been established.	1 published mouse study; early immunogenicity evidence
**Chimeric vaccine displaying CVA16 and CVA10 epitopes on an EV-A71 scaffold** *Chimeric VLP*	Induces antibodies specific to EV-A71, CVA16, and CVA10. After challenge in mice, protection rates against CVA16 and CVA10 are 20% and 40%, respectively [56].	Heterologous protection is weak and unbalanced, indicating that conformational epitope presentation and/or immunodominance require further optimization.	1 published mouse study; early antigen-design proof of concept

Note: Study counts refer to distinct primary studies cited for each candidate; clinical-trial registry entries are reported separately from published studies.

**Table 2:** Proposed stage-gated framework for antigen inclusion in multivalent HFMD vaccines in China.

Decision Gate	Evidence Required	Criteria to Proceed	Criteria to Defer or Revise
**1. Product objectives and target populations**	Prespecified use, target age, immunization schedule, baseline immune status, and prior EV-A71 vaccination [12,15,84,85,86]	The added component is expected to improve a clearly defined target outcome.	The component only broadens theoretical coverage or is unlikely to improve the prespecified outcome.
**2. Epidemiological need and persistence**	Pathogen-confirmed type-specific incidence, hospitalizations, severe disease, and outbreak data spanning multiple consecutive epidemic seasons, with consideration of epidemic cycles and surveillance changes [7,21,40,45,50,52]	The type causes a sustained, measurable absolute disease burden.	The rationale rests mainly on a short-term increase in proportional detection, a single localized outbreak, or unstable surveillance findings.
**3. Antigenic coverage and candidate-strain suitability**	Neutralization coverage of contemporary representative isolates and major circulating lineages, including effects of assay strain and structural antigenicity [55,81,82,87,88]	The candidate reproducibly covers the major circulating lineages.	Coverage is narrow, highly assay-strain dependent, or poorly matched to circulating viruses.
**4. Manufacturing and component-specific quality control**	Reproducible manufacturing and component-specific methods for identity, content, conformation, potency, and stability [17,18,19,34,76,77,80,83,89]	Each component can be manufactured reproducibly, alone and in combination, while retaining functional antigenic activity.	Yield or stability is inadequate, components are formulation-incompatible, or component-specific potency methods are lacking.
**5. Immune-response balance and dose optimization**	Comparison with corresponding monovalent or lower-valency formulations for antibody titers, seroconversion, dose–response relationships, and durability [28,31,66,67,73,78,84]	All components meet prespecified response criteria without materially weakening responses to existing components.	Responses remain imbalanced, require impractical doses or schedules, or show important immune interference.
**6. Clinical evaluability and development strategy**	Feasibility of accruing type-specific cases, appropriate trial settings and epidemic seasons, pathogen-confirmed endpoints, and an immunobridging rationale [15,16,21,28,31,40]	Interpretable component-specific or pooled efficacy against covered types can be obtained.	Incidence is too low or unstable, and no credible correlate of protection or alternative evaluation strategy is available.
**7. Incremental public-health value and implementation feasibility**	Reduction in cases and severe disease relative to a lower-valency comparator, together with price, achievable coverage, duration of protection, and implementation conditions [14,90,91,92,93]	Additional health benefit remains meaningful after accounting for cost and vaccination complexity.	The added component prevents little disease while substantially increasing price, reducing uptake, or complicating implementation.

Note: This framework is intended to support comparative research-and-development decisions and is not a formal regulatory scoring tool or clinical vaccination recommendation. A “Go” decision indicates support for progression to the next development stage and does not imply that a component should be included universally across all regions. “Defer or revise” may involve generating additional evidence, restricting development to selected high-burden settings, modifying the candidate strain or dose/formulation, or temporarily excluding the component. Valency selection should be reassessed as pathogen circulation, vaccine coverage, duration of protection, and programmatic costs evolve. For application beyond China, the framework should be recalibrated to local enterovirus epidemiology, vaccination patterns, surveillance capacity, and implementation conditions rather than transferring China-specific priorities directly.

## Data Availability

No new data were created or analyzed in this study. Data sharing is not applicable to this article.
